# Identification and prognostic value of a glycolysis-related gene signature in patients with bladder cancer

**DOI:** 10.1097/MD.0000000000023836

**Published:** 2021-01-22

**Authors:** Zhengyuan Wu, Zhenpei Wen, Zhengtian Li, Miao Yu, Guihong Ye

**Affiliations:** a Department of Orthopedics Trauma and Hand Surgery; b Department of Bone and Joint Surgery; c Departments of Pathology; d Departments of Ultrasound, The First Affiliated Hospital of Guangxi Medical University, Nanning, China.

**Keywords:** bladder cancer, glycolysis, prognosis, risk score, survival

## Abstract

Bladder cancer (BC) is one of the most common malignancies worldwide. Several biomarkers related to the prognosis of patients with BC have previously been identified. However, these prognostic models use only one gene and are thus not reliable or accurate enough. The purpose of our study was to develop an innovative gene signature that has greater prognostic value in BC. So, in this study, we performed mRNA expression profiling of glycolysis-related genes in BC (n = 407) cohorts by mining data from The Cancer Genome Atlas (TCGA) database. The glycolysis-related gene sets were confirmed using the Gene Set Enrichment Analysis (GSEA). Using Cox regression analysis, a risk score staging model was built based on the genes that were determined to be significantly associated with BC outcome. Eventually, the system of risk score was structured to predict a patient's survival, and we identified four genes (*CHPF*, *AK3*, *GALK1*, and *NUP188*) that were associated with the outcomes of BC patients. According to the above-mentioned gene signature, patients were divided into two risk subgroups. The analysis showed that our constructed risk model was independent of clinical features and that the risk score was a highly powerful tool for predicting the overall survival (OS) of BC patients. Taking together, we identified a gene signature associated with glycolysis that could effectively predict the prognosis of BC patients. Our findings offer a new perspective for the clinical research and treatment of BC.

## 1 Introduction

Bladder cancer (BC) is the most common malignancy of the urinary tract and the 10th most common cancer around the world. It was estimated that 549,000 new cases of cancer and 200,000 deaths were attributed to BC in 2018. Men are four times more likely to be affected than women and the global cancer incidence and mortality rate are 9.6 and 3.2 per 100,000 men, respectively.^[1]^ Nearly 75% of BC patients have non-muscle-invasive BC (NMIBC), which has a common low-grade, papillary morphology.^[2,3]^ The remaining 25% of the patients can have high-grade, muscle-invasive BC (MIBC) of a non-papillary morphology that disseminates regionally and/or systemically.^[3,4]^ In clinical practice, the prognosis of BC often depends on BC histopathology.^[5]^ However, the prognosis is not exact and only provides a simple stratification of risk. Furthermore, there are differences between individuals. Patients with the same histopathology might also have variable outcomes.

At present, the detection methods of BC mainly include cystoscopy, urinary cytology, and imageology. There are still challenges for the detection of BC in patients without hematuria, and small lesions in an incompletely filled bladder are difficult to detect by computerized tomography (CT) and ultrasound (US). In low-grade BC, the detection sensitivity of urinary cytology is as low as 16%.^[6,7]^ Nevertheless, to improve the overall survival (OS) of patients with BC, it is thus virtually to diagnosis of BC at an early stage. Hence, effective and minimally invasive methods to identify risk groups of BC are always required.^[4]^ In recent years, metabolomics involving glycolysis and beta-oxidation is an emerging field for the investigation of biochemical processes.^[8–10]^ A popular area of study is the Warburg effect which showed that the manner in which cancer cells metabolize glucose is distinct from that of cells in normal tissues. Cancer cells prefer to “ferment” glucose to lactate even in the availability of oxygen. Thus, this metabolism is known as “aerobic glycolysis.”^[11,12]^ The combination of novel molecular biomarkers linked with glycolysis and the existing prognostic methods may, therefore, be a viable strategy to enhance the detection and prognosis of BC.

Currently, several biomarkers associated with BC prognosis have been explored. PFKFB4 is significantly overexpressed in patients with late-stage carcinoma and predicts the progression of multiple tumors.^[13]^ Moreover, FOXJ1 is upregulated in BC cells and increases cellular proliferation by enhancing glycolysis and is associated with poorer outcomes.^[14]^ Nevertheless, predicting BC prognosis with a single gene is not as accurate as when a combination of biomarkers is used. Multigene glycolysis-related prognostic signatures can provide fresh insight into the clinical study and individual treatment of BC. Therefore, the establishment of an expression-based gene signature is vital for determining the prognosis of BC patients.

The glycolysis-related gene sets were confirmed by using Gene Set Enrichment Analysis (GSEA) in this study. Using GSEA, we selected the gene sets that showed statistical significance and concordant differences in the relevant biological processes. As it was difficult to analyze and annotate the results, the Molecular Signatures Database (MSigDB) integrated with GSEA was designed to allow the annotation of the gene sets. From the GSEA, we developed the hallmark gene sets associated with glycolysis. The glycolysis-related genome expressions of 407 samples of BC were extracted for further analysis. A total of 171 significant mRNAs were identified to be significantly related to glycolysis. Furthermore, we established a glycolysis-related prognostic signature, comprising four genes, which can effectively predict the survival of BC patients. In addition, the prognostic advantage of risk score, compared with other clinical features, was also demonstrated in BC patients by a series of bioinformatics analyses.

## 2 Materials and methods

### 2.1 Clinical data and mRNA expression profiles of patients

The transcriptome profiling of mRNA and clinical data of BC patients were extracted from the TCGA database (https://cancergenome.nih.gov/). Approval by the Ethics Committee was not necessary because all data were collected from publicly available databases (TCGA). In this study, a total of 412 patients with clinical features (age, gender, survival status, grade, TNM stage, T stage, N stage, and M stage) were included. The clinical features of BC patients are shown in Table 1.

**Table 1:** Clinical features of bladder cancer patients (n = 412) from TCGA database.

Variables	Patients, n (%)	Variables	Patients, n (%)
Survival status		T stage	
Dead	159 (38.6)	T0	1 (0.2)
Alive	253 (61.4)	T1	3 (0.7)
Gender		T2	120 (29.2)
Male	108 (26.2)	T3	196 (47.6)
Female	304 (73.8)	T4	59 (14.3)
Age (years)		TX	1 (0.2)
≦65	162 (39.3)	Unknown	32 (7.8)
>65	250 (60.7)	M stage	
Grade		M0	196 (47.6)
High	388 (94.2)	M1	11 (2.7)
Low	21 (5.1)	MX	202 (49.0)
Unknown	3 (0.7)	Unknown	3 (0.7)
Stage		N stage	
I	2 (0.5)	N0	239 (58.0)
II	131 (31.8)	N1	47 (11.4)
III	141 (34.2)	N2	76 (18.5)
IV	136 (33.0)	N3	8 (1.9)
Unknown	2 (0.5)	NX	36 (8.7)
		Unknown	6 (1.5)

### 2.2 Gene set enrichment analysis

GSEA was used to determine if the glycolysis-related function differences between the normal sample and BC samples are statistically significant.^[15]^ The expression of 56753 mRNAs, derived from the TCGA dataset, were analyzed. The functions investigated for further analysis were determined using normalized *P*-values (*P* < .05).^[16]^

### 2.3 Statistical analysis

The 56753 mRNA expression profiles were presented as initial data, and glycolysis-related genes were selected by GSEA. Each gene expression data was log2 transformed for the following analysis. After removing the samples with unknown and missing values, the clinical samples of 407 BC patients were selected for further study. The genes significantly associated with OS were identified by univariate Cox regression analysis (*P* < .05).

The filtered genes entered into the next multivariate Cox proportional regression model. The final glycolysis-related genes and prognostic model were identified by the evaluation of survival factors effect. With the function coxph of R package, we constructed a survival risk score model,^[17,18]^ and it is expressed by the following formula:

Risk score = β1 × expression of gene 1 + β2 × expression of gene 2 + … + βn × expression of gene n.

BC patients were separated into high-risk and low-risk subgroups using the median risk score. The prognostic significance of the risk score was then validated by the log-rank test and survival curves. We also applied Student's *t* test to determine if the gene expression levels of normal and BC samples were significantly different. The cBioPortal for Cancer Genomics (http://www.cbioportal.org/) was used to study the alterations of genes related to prognosis. All data were analyzed using SPSS 16.0 and GraphPad Prism 7.0 software.

## 3 Results

### 3.1 Preliminary screening of glycolysis-related genes sets

**Figure 1:**
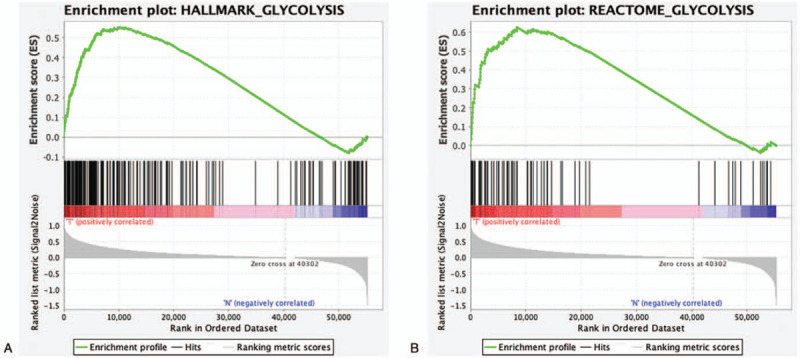
Enrichment plots of two glycolysis-related gene sets in BC from GSEA. The gene sets of HALLMARK_GLYCOLYSIS (A) and REACTOME_GLYCOLYSIS (B) differ significantly between normal samples and BC samples.

The expression levels of 56753 mRNA and the clinical data of 407 patients were extracted from the TCGA database. We downloaded seven hallmark gene sets that represent glycolysis-related biological processes from MSigDB—HALLMARK_GLYCOLYSIS, REACTOME_GLYCOLYSIS, BIOCARTA_FEEDER_PATHWAY, BIOCARTA_GLYCOLYSIS_PATHWAY, KEGG_GLYCOLYSIS_GLUCONEOGENESIS, MODULE_306, and REACTOME_REGULATION_OF_GLYCOLYSIS_BY_FRUCTOSE_2_6_BISPHOSPHATE_METABOLISM. The abovementioned data was used to explore the statistical differences between the normal and BC groups by GSEA. Gene sets named HALLMARK_GLYCOLYSIS and REACTOME_GLYCOLYSIS were found to be statistically significant (Table 2, Fig. 1A and B). Both gene sets were used in further studies.

**Table 2:** Gene sets enriched in BC.

GS follow link to MSigDB	SIZE	NES	NOM*P*	FDR*q*
HALLMARK_GLYCOLYSIS	199	1.7423	.0016	0.0016
REACTOME_GLYCOLYSIS	71	1.7037	.0138	0.0138
BIOCARTA_FEEDER_PATHWAY	9	−0.9864	.4472	0.4472
BIOCARTA_GLYCOLYSIS_PATHWAY	3	1.0411	.4730	0.4730
KEGG_GLYCOLYSIS_GLUCONEOGENESIS	62	1.0031	.4545	0.4545
MODULE_306	26	1.2720	.2451	0.2451
REACTOME_REGULATION_OF_GLYCOLYSIS_BY_FRUCTOSE_2_6_BISPHOSPHATE_METABOLISM	12	1.2862	.1652	0.1652

### 3.2 Identification of glycolysis-related genes associated with OS and prognostic model construction in BC patients

In this study, we used the Cox regression model method to identify genes associated with the OS of BC patients. The two glycolysis-associated gene sets were analyzed using the univariate Cox regression analysis. The results showed that a total of 7 genes (*ENO1*, *SLC16A3*, *CHPF*, *AK3*, *PLOD1*, *GALK1*, and *NUP188*) had statistical significance (*P* < .05). Subsequently, a total of 4 genes (*CHPF*, *AK3*, *GALK1*, and *NUP188*) (Table 3) were selected by the multivariate Cox regression analysis to have prognostic value. The corresponding risk scores were calculated to determine the prognosis of each BC patient.

**Table 3:** Details of the four selected mRNAs.

mRNA	Ensemble ID	Chromosome location	β(Cox)	HR	*P*
CHPF	ENSG00000123989	chr2:219538947–219543787	0.156689	1.169632	.0179
AK3	ENSG00000147853	chr9:4709559–4742043	−0.40384	0.667749	.0038
GALK1	ENSG00000108479	chr17:75751594–75765711	0.350739	1.420117	.0002
NUP188	ENSG00000095319	chr9:128947699–129007096	0.444476	1.559673	.0084

**Figure 2:**
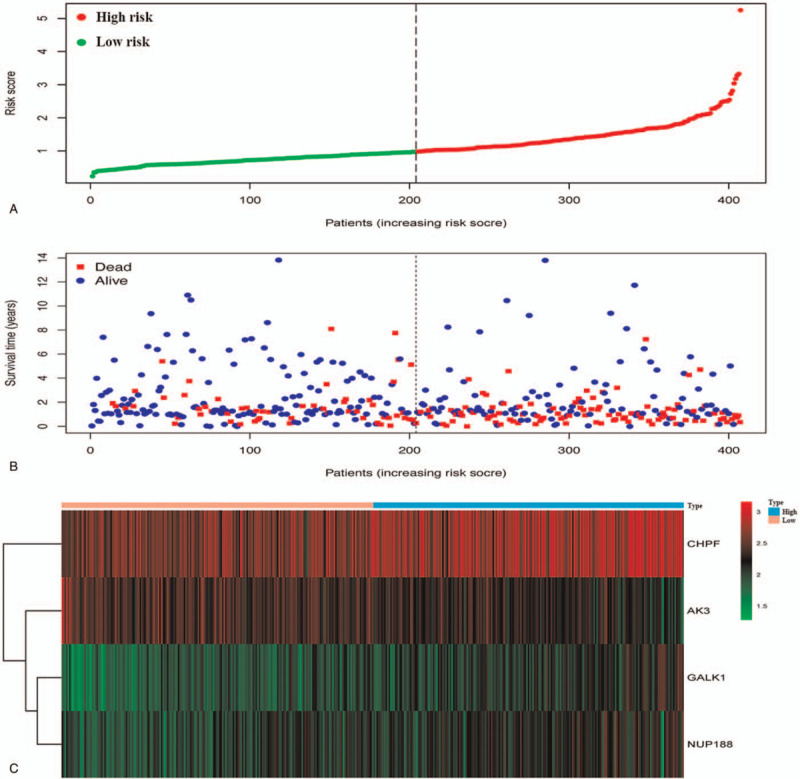
The four-gene risk signature related to glycolysis predicts the OS of BC patients. (A) Risk score curve of patients’ distribution. (B) The relationship between survival status and survival time (years). (C) The alteration of genes expression profile is associated with risk score in Heat map.

The median risk score, computed by the above formula, was used as a threshold to divide BC patients into high-risk (n = 204) and low-risk groups (n = 203) (Fig. 2A). The low-risk group had a longer survival time than the high-risk group had (Fig. 2B). The heatmap showed that the three genes (*CHPF*, *GALK1*, and *NUP188*) were significantly upregulated and had higher risk scores, whereas the fourth gene (*AK3*) was downregulated (Fig. 2C).

### 3.3 Univariate and multivariate analyses of independent prognostic factors

**Figure 3:**
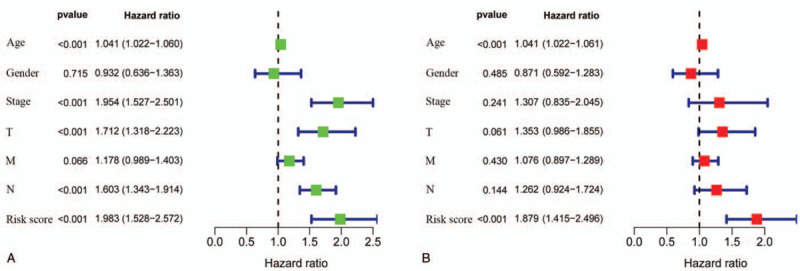
Univariable and multivariable independent prognostic analyses for clinical features in BC. (A) The age, TNM stage, T stage, N stage, and risk score had significant differences (*P* < .05) in the univariate analysis, which indicate those factors were related to patients OS. (B) In the multivariate analysis, the age and risk score could be selected as the independent prognostic factors with *P* values <.05.

The OS-related variables in the univariate and multivariate Cox proportional hazard models are shown in Table 4, and Figure 3A and B. In the univariate analysis, age (HR, 1.041; 95% CI, 1.002–1.060; *P* < .001), TNM stage (HR, 1.954; 95% CI, 1.527–2.501; *P* < .001), T stage (HR, 1.712; 95% CI, 1.318–2.223; *P* < .001), N stage (HR, 1.603; 95% CI, 1.343–1.914; *P* < .001), and risk score (HR, 1.938; 95% CI, 1.528–2.572; *P* < .001) were found to be correlated with poor OS. According to the multivariate analysis, age (HR, 1.041; 95% CI, 1.022–1.061; *P* < .001) and risk score (HR, 1.879; 95% CI, 1.415–2.496; *P* < .001) could independently impact OS. These results declared that our risk score could also be regarded as an independent prognostic factor for BC patients.

**Table 4:** Univariable and multivariable analyses for each clinical feature.

Clinical feature	Univariate analysis			Multivariate analysis		
	HR	95%CI of HR	*P*	HR	95%CI of HR	*P*
Age	1.041	1.022–1.060	<.01	1.041	1.022–1.061	<.001
Gender	0.932	0.636–1.363	.715	0.871	0.592–1.283	.485
Stage	1.954	1.527–2.501	<.001	1.307	0.835–2.045	.241
T	1.712	1.318–2.223	<.001	1.353	0.986–1.855	.061
M	1.178	0.989–1.403	.066	1.076	0.897–1.289	.430
N	1.603	1.343–1.914	<.001	1.262	0.924–1.724	.144
Risk score	1.983	1.528–2.572	<.001	1.879	1.145–2.496	<.001

### 3.4 Genetic alteration and differential expression analysis of the prognostic model

**Figure 4:**
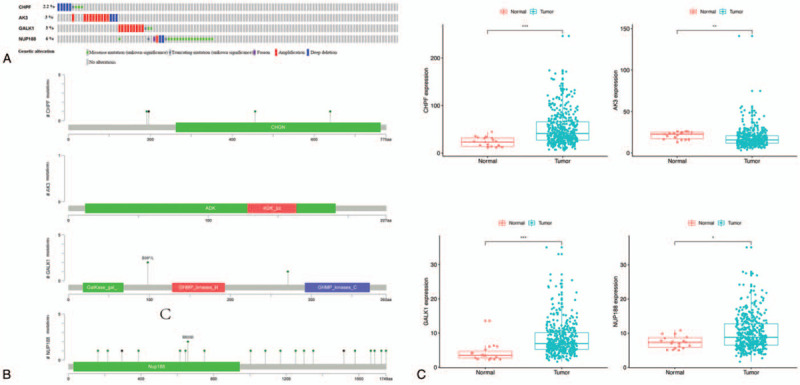
Genetic alteration and the identification of genes signature associated with patient's prognosis. (A) The mutations frequency and type of four glycolysis-related genes in BC clinical samples. (B) The amount, height and location of the annotation represent different mutations of each gene. (C) The expression of selected genes was different in normal and tumor groups (^∗^*P* < .05, ^∗∗^*P* < .01, ^∗∗∗^*P* < .001).

The cBioPortal was used to determine the variation of the screened genes in BC cases. The results showed that the total frequency of genetic alteration was 14.2%. The genetic alterations and mutations are shown in Figure 4A and B. The alterations are noted using different colors. Furthermore, we investigated the expression levels of the four selected genes in healthy controls and BC groups (Fig. 4C). The results showed that the expression levels of CHPF, GALK1, and NUP188 were significantly increased in BC patients, while those of AK3 was significantly decreased compared to those in healthy controls. This result was highly consistent with the expression levels of the mRNA in the two risk subgroups, which indicates consistency between the BC prognosis and the mRNA expression of these four genes.

### 3.5 Validation of the predictive value of four-mRNA signature in BC prognosis

**Figure 5:**
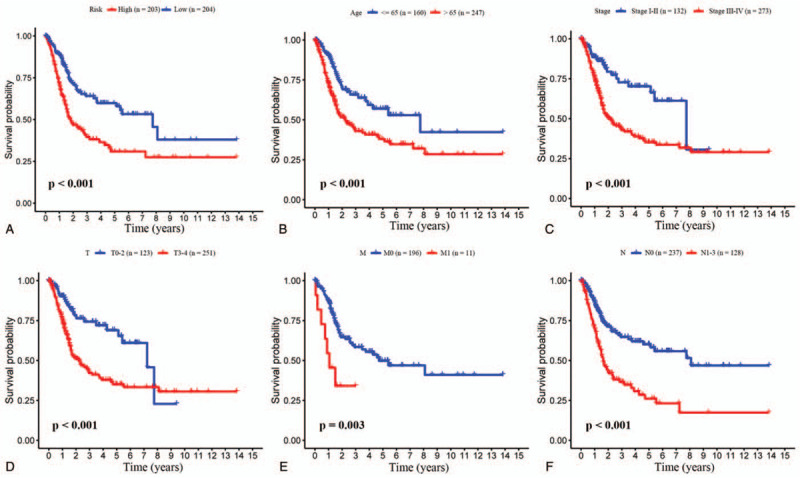
Kaplan–Meier analysis for BC patients. According to risk score, individuals with BC divided into the high-risk and low-risk groups, and Kaplan–Meier OS curve showed significantly statistical difference in two groups (A). Clinical features that include age (B), TNM stage (C), T stage (D), M stage (E), and N stage (F) were also significantly associated with patients OS.

The survival curves showed that clinical features, including high-risk score, age (age >65), T stage (T3 and T4), M stage (M1), N stage (N1, N2, and N3), and TNM stage (III and IV), were significantly related to poor OS of patients with BC (Fig. 5A–F).

**Figure 6:**
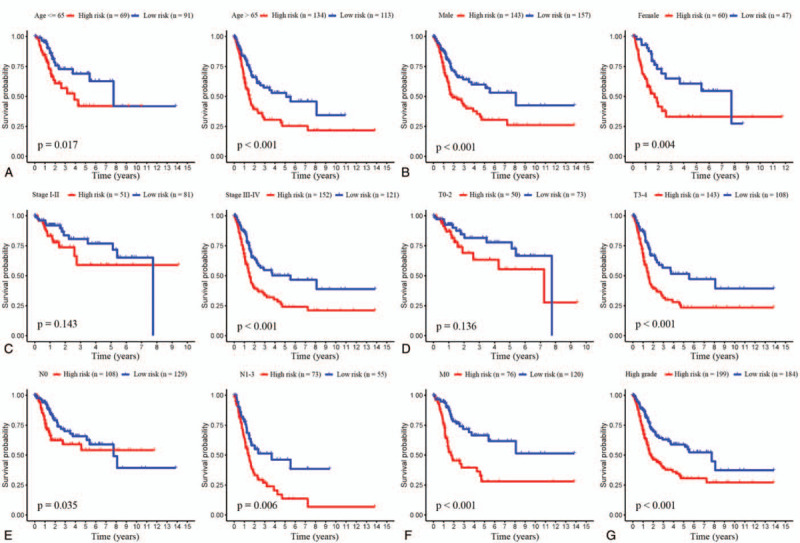
Survival analysis of Kaplan–Meier curves with clinical subgroups for the prognostic value of risk signature. (A) K–M curves for the patient group with age ≤65 (n = 160) and patient group with age >65 (n = 247). (B) K–M curves for the male group (n = 300) and female group (n = 107). (C) K–M curves for the stage I to II group (n = 132) and stage III to IV group (n = 273). (D) K–M curves for the T0–2 group (n = 123) and the T3–4 group (n = 251). (E) K–M curves for the N0 group (n = 237) and the N1–3 group (n = 128). K–M curves for the M0 group (n = 196) (F) and the high-grade group (n = 383) (G).

Furthermore, the results in Figure 6A, B, and E suggest that the risk score had a superior prognostic capacity for BC patients who were classified by age (age ≤65 or >65), gender (male or female), and N stage (N0 or N1, N2, N3). Regardless, when patients were stratified into various subgroups based on TNM and T stages, the risk score played a different role. The predictive value of risk score varied among BC patients who were divided into subgroups based on TNM and T stages (Fig. 6C and D). The survival probability of the patients with stage I–II (*P* = .143) and stage T0–2 (*P* = .136) were not significantly different between the high-risk and low-risk groups. In contrast, statistically significant differences between the prognosis value of risk scores were revealed in stage III–IV and stage T3–4 subgroups (*P* < .001). However, results of the small sample size in patients with M1 stage (n = 11) and low-grade (n = 21), the prognostic value of the risk score was only detected in the patients with M1 stage and high-grade subgroup. The patients in the high-grade subgroup with high risk also had significantly shorter OS (*P* < .001) (Fig. 6G). Similarly, there was a shorter OS in the M0 stage high-risk subgroup (*P* < .001) (Fig. 6F). Our findings indicated that the prognostic model was effective in the prognosis of different clinical subgroups and applied especially well for the late stages of the disease.

## 4 Discussion

With the development in the studies on energy metabolism, researchers are beginning to realize that energy metabolism and malignancies are closely linked. There are also multiple studies that focus on glycolysis and cancer. According to the findings of Warburg et al, the metabolism of tumor cells shifts from oxidative phosphorylation to glycolysis in the presence of oxygen. This is known as the “Warburg effect” or “aerobic glycolysis”^[11]^ and is essential for tumor growth and proliferation. However, the advantages of aerobic glycolysis in cancer are still in debate. The major argument lies in the following two points: first, cancer cells tend to produce ATP to maintain energy supply through a non-economical glycolysis pathway rather than the oxidative phosphorylation pathway^[19]^; second, even under aerobic conditions, persistent glycolysis metabolism is also an adaptation to intermittent hypoxia in pre-malignant lesions. With the upregulation of glycolysis, cancer cells develop phenotypes that can tolerate acid-induced cell toxicity in microenvironmental acidosis.^[20]^ Moreover, aerobic glycolysis increases the uptake of nutrients, elevate flux through biosynthetic pathways, and maintain high levels of glycolytic intermediates to support anabolic reactions in cancer cells.^[21]^ Currently, there are several studies focusing on cancer progression and glycolysis. However, research determining the prognostic value of glycolysis-related genes set is still limited. We hence try to construct a risk score staging model from several glycolysis-related genes to improve the prediction efficiency of OS in BC patients.

GSEA is a computational method that can identify the statistical significance of prior defined gene set and concordant differences between two biological states. In our study, the GSEA was performed to screen glucose-related gene sets of BC patients. By analyzing the NES and P-value, HALLMARK_GLYCOLYSIS and REACTOME_GLYCOLYSIS gene sets were selected for further analysis. Furthermore, four genes *CHPF*, *AK3*, *GALK1*, and *NUP188* were identified as hub glycolysis-associated genes which were significantly connected with the overall survival of BC patients. Among these genes, CHPF was identified to be engaged in chondroitin polymerization. CHPF exerts dual GlcAT-II and GalNAcT-II activity. Moreover, CHPF plays a critical role in the biosynthesis of chondroitin sulfate through co-operation with CSS3 or ChSy-1.^[22,23]^ These findings indicate that CHPF is involved in energy metabolism and may be related to glycolysis. AK3, arginine kinase 3, located on chromosome 9, functions mainly in the mitochondrial matrix and is involved in the homeostasis of adenine nucleotide composition in various organisms. Moreover, AK3 has been demonstrated to have anticancer effect.^[24,25]^ Qin et al showed that the expression level of AK3 was downregulated in breast cancer patients and that decreased AK3 level was significantly associated with poor OS. In hepatocellular carcinoma, AK3 is also significantly downregulated, and the AK3-encoded protein has been identified as a specific biomarker to detect hepatocellular carcinoma.^[24,26]^ Interestingly, the expression of AK3 was also significantly downregulated in our study. GALK1 encodes galactokinase (GALK). Mutations in GALK1 can cause GALK deficiency or galactosemia type 2. In addition, individuals with GALK deficiency cannot phosphorylate galactose and consequently accumulate galactose and galactitol.^[27,28]^ These findings indicate that GALK1 also plays a role in metabolism. Furthermore, GALK1 is a novel therapeutic target for HCC. Tang et al reported that GALK1 siRNAs could effectively inhibit the growth of HepG2 cells.^[29]^ However, the relationship between GALK1 and BC is still unclear. NUP188 encodes nucleoporin, a component of the nuclear pore complex (NPC). Nucleoporin regulates chromosome segregation in mitotic cells by promoting chromosome alignment. The aneuploidy in some cancer cells could be caused by defects in the chromosomal segregation process.^[30]^ NUP188 may thus play a role in oncogenesis and the proliferation of cancer cells. The relationship between NUP188, BC, and glycolysis mechanism is still unclear and needs further exploration.

Moreover, to identify whether these specific genes could be used as a prognostic factor in BC, we constructed a novel prognostic prediction model based on the four hub genes. The results of univariate and multivariate Cox regression analyses indicated that we found a novel molecular biomarker—a glycolysis-related risk signature—that can be used to accurately predict clinical outcomes of BC patients. We then verified it by using K–M analysis and observed that patients with high-risk scores had significantly poorer OS. These results showed that the four-gene risk score had high prognostic value and can not only serve as a new method for predicting the prognosis of BC patients but also assist clinicians in formulating personalized therapies.

Nonetheless, this study has some limitations. First, this study was purely based on computational data and designed as a retrospective analysis; more prospective research should be performed to verify our results. Second, our results lack in vitro or in vivo validation to confirm the reliability of the proposed mechanisms. Therefore, we need to conduct several experiments to prove the mechanistic connections between these genes and BC progression as warranted.

## 5 Conclusion

In conclusion, we identified four glycolysis-related genes that were significantly associated with the overall survival of BC patients. The four-gene signature was independent of other standard factors that could predict the outcome of BC patients. Combined with the existing methods, the application of this gene signature could potentially benefit the treatment and management of BC. Moreover, our results also indicated that together, the four glycolysis-related genes, *CHPF*, *AK3*, *GALK1*, and *NUP188*, form a promising prognostic biomarker that could offer insights for the clinical research and treatment of BC.

## Author contributions

**Conceptualization:** Guihong Ye.

**Data curation:** Zhengyuan Wu.

**Formal analysis:** Zhengyuan Wu, Miao Yu.

**Methodology:** Zhengtian Li.

**Project administration:** Guihong Ye.

**Writing – original draft:** Zhengyuan Wu, Zhenpei Wen.

**Writing – review & editing:** Guihong Ye.
